# Luteolin Inhibits Dexamethasone‐Induced Osteoporosis by Autophagy Activation Through miR‐125b‐5p/SIRT3/AMPK/mTOR Axis, an In Vitro and In Vivo Study

**DOI:** 10.1002/fsn3.70071

**Published:** 2025-03-17

**Authors:** Liang Tang, Xinyu Fan, Yongqing Xu, Yeming Zhang, Gang Li

**Affiliations:** ^1^ Department of Geriatrics The First People's Hospital of Yunnan Province (The Affiliated Hospital of Kunming University of Science and Technology) Kunming China; ^2^ Orthopedics 920th Hospital of Joint Logistics Support Force Kunming China; ^3^ Orthopedics The People's Hospital of Xiangyun County Xiangyun China

**Keywords:** autophagy, luteolin, miRNA, osteoprosis, SIRT3

## Abstract

Luteolin (LUT) has been suggested as an inhibitor of osteoporosis (OP). This investigation examines the pivotal role of the miR‐125b‐5p/SIRT3/AMPK/mTOR pathway in mediating luteolin‐induced effects on OP. Mesenchymal stem cells derived from bone marrow (BMSCs) were exposed to dexamethasone (DEX) to create an in vitro model of OP. Following treatment with luteolin, the levels of miR‐125b‐5p and SIRT3 were quantified using reverse transcription polymerase chain reaction. Moreover, SIRT3, AMPK, mTOR protein levels, and osteogenesis (OPN, Runx2, OSX, and OCN), and autophagy (p62, ATG5, LC3, and BECN1) were evaluated using ELISA. Additionally, specific mimics and siRNA were constructed to overexpress miR‐125b‐5p or downregulate SIRT3. Furthermore, animal models of DEX‐induced OP were constructed to assess the effects of LUT at doses of 50 and 100 mg/kg/day on bone histology, stereology, biochemistry, and the expression of the miR‐125b‐5p, SIRT3/AMPK/mTOR axis, and markers of osteogenesis and autophagy. The findings revealed that LUT suppressed miR‐125b‐5p expression, overexpressed SIRT3 and AMPK, and downregulated mTOR in BMSCs compared to DEX (*p*‐value < 0.01). Interestingly, LUT restored the levels of markers for osteogenesis and autophagy (*p*‐value < 0.001). The overexpression of SIRT3 or miR‐125b‐5p downregulation inhibited LUT therapeutic properties. In animals, LUT improved bone histology (*p*‐value < 0.05) and inhibited miR‐125b‐5p and mTOR expression while overexpressing SIRT3 and AMPK (*p*‐value < 0.001). RUNX2, OSX, OPN, and OCN levels were improved, and autophagy was enhanced in LUT‐treated rats. The current findings revealed that LUT could promote osteogenesis and improve OP via autophagy activation through the miR‐125b‐5p/SIRT3/AMPK/mTOR pathway.

## Graphical abstract

The present findings revealed that osteoporosis is associated with the overexpression of miR‐125b‐5p and alteration in the SIRT3/AMPK/mTOR axis, which is followed by the suppression of autophagy in BMSCs and bone tissue. Meanwhile, the administration of luteolin was able to improve the bone tissue of animals treated with DEX according to histological, biochemical, and molecular analyses. The change in the expression of miR‐125b‐5p and the SIRT3/AMPK/mTOR axis, which was accompanied by the induction of autophagy, could be considered the underlying mechanism of the therapeutic properties of luteolin in confronting osteoporosis. Nonetheless, the current findings cannot be assumed as evidence for the application of luteolin as a therapeutic strategy in patients with osteoporosis, since further studies, especially clinical trials, are necessary.

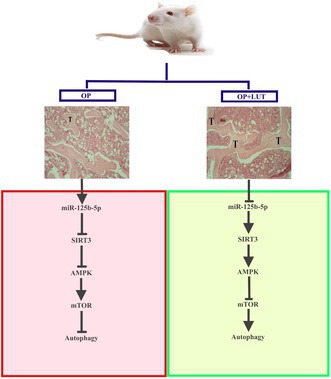

## 1 Background

Osteoporosis (OP), the most prevalent degenerative skeletal disease, is a chronic disorder millions of people around the world suffer from (Abo‐Elenin et al. 2024). The diagnosis of OP is often challenging as the disease is mainly asymptomatic and is diagnosed when the fracture occurs (Aibar‐Almazán et al. 2022). Moreover, OP is considered a complicated disease since a variety of causes such as lifestyle, hormonal imbalances, nutritional deficiencies, and genetic predispositions could play a role in the occurrence and progression of OP (Aibar‐Almazán et al. 2022). Recent evidence indicates that osteogenesis, a process that often occurs to confront bone depletion caused by OP, is mediated by stem cells derived from bone marrow (BMSCs) and through mediators such as runt‐related transcription factor 2 (RUNX2, transcription factor related to osteoblast differentiation), osterix (OSX, also a transcription factor downstream of RUNX2 that promotes osteoblast differentiation), osteopontin (OPN, an extracellular matrix protein that transfers the mechanical stress signal to osteoblasts), and osteocalcin (OCN, a marker for mature osteoblasts) (Gao et al. 2015; Gromolak et al. 2020; Mollentze et al. 2021; Sun et al. 2012). In fact, in dealing with OP, BMSCs are activated and through mediators such as exosomes, they modulate the gene expression participating in osteogenesis to improve the osteoblast's activity and prevent the osteoclast's activity (Zhang, Jiao, et al. 2020). Meanwhile, upstream regulators have been investigated to manipulate bone cell activity and suggest potential therapeutic strategies. MicroRNAs (miRNAs) have been widely assumed to be the main regulators of intracellular signaling that control cell activity through changes in gene expression (Hensley and McAlinden 2021; Laxman et al. 2015).

Mir‐125b is considered a crucial microRNA that significantly influences osteoblast differentiation by modulation of different pathways, hence contributing to the OP progression (Ogasawara et al. 2024). Sirtuin 3 (SIRT3) is one of the most important targets of miRNAs (Li and Lu 2023; Yang et al. 2024), which modulates the gene expression engaged in autophagy, including beclin‐1 (BECN1), autophagy‐related protein 5 (ATG5), microtubule‐associated protein 1A/1B‐light chain 3 (LC3), and sequestosome‐1 (SQSTM1/p62), through the upstream mediators of the autophagic flux (e.g., mammalian target of rapamycin [mTOR] and AMP‐activated protein kinase [AMPK]) (Chen et al. 2024). Indeed, targeting the regulators of autophagic flux in bone remodeling is extremely important as autophagy is recognized as both a determinant of cell survival and a catabolic pathway that participates in providing materials/energy necessary for construction within the cell (di Giacomo et al. 2020; Samare‐Najaf, Neisy, et al. 2023; Samare‐Najaf, Samareh, et al. 2023; Zhu et al. 2022).

Although estrogen administration as a type of hormone replacement therapy is considered the main OP treatment strategy, particularly in postmenopausal OP, long‐term safety and efficacy remain crucial concerns of conventional therapeutic approaches (Walker and Shane 2023). Recent investigations focus on herbs as promising complementary therapies for OP treatment due to the native pharmacological properties of these phytochemicals (Karimi et al. 2024). Luteolin (LUT) is a flavonoid phytochemical with favorable properties in the alleviation of a wide range of chronic disorders (for instance cancer, diabetes, etc.) (Abdrabou et al. 2024; Huang et al. 2023). Recently, it has been shown that LUT may represent anti‐osteoporosis features by activation of PI3K‐AKT signaling followed by suppression of pyroptosis, a type of regulated cell death, in osteoblasts (Chai et al. 2024). Moreover, evidence of LUT's ability to change the pathways affecting OP, such as the SIRT3/AMPK/mTOR axis and downstream autophagy, has been reported (Vongthip et al. 2024; Wu et al. 2023; Yuan et al. 2023). However, the ability of LUT to improve OP and the mechanism by which it provides therapeutic effects have not been elucidated.

Osteoporosis continues to be a significant global health issue, prompting extensive research into the molecular mechanisms that drive its progression, with the aim of developing novel therapeutic approaches. Recent findings have identified miR‐125b‐5p and the SIRT3/AMPK/mTOR signaling pathway as potential targets for addressing OP through their influence on autophagy. Additionally, LUT has demonstrated properties that may counteract OP and modulate various pathways implicated in the disease. Consequently, this study sought to evaluate the effectiveness of LUT in both cellular and animal models of OP, while also exploring the underlying mechanisms involved. To achieve this, in vitro and in vivo models of OP were induced using dexamethasone (DEX), allowing for an assessment of the interplay between miR‐125b‐5p and the SIRT3/AMPK/mTOR axis. Moreover, the therapeutic potential of LUT was analyzed using knockdown and overexpression assays and spectrometry, RT‐PCR, ELISA, and histopathological approaches.

## 2 Materials and Methods

### 2.1 Chemicals

Thermo Fisher (Waltham, MA, USA) provided TRIzol reagent, fetal bovine serum (FBS), Dulbecco's modified Eagle medium (DMEM), alpha minimum essential medium (α‐MEM), streptomycin, penicillin, trypsin‐ethylene diamine tetraacetic acid (EDTA), and protease inhibitor tablets. Cell culture flasks and plates were Obtained from Costar (Tewksbury, MA, USA). Sigma‐Aldrich Supplied (St. Louis, MO, USA) luteolin, L‐ascorbic acid, nicotinamide, phosphate‐buffered saline (PBS), sirtinol, ethanol (EtOH), paraformaldehyde, DEX, 2′,7′‐dichlorofluorescein diacetate (DCFH‐DA), and β‐glycerol phosphate. ELISA kits were purchased for the measurement of SIRT3 (Catalogue number: abx156091, abbexa, UK), AMPK (Catalogue number: NBP3‐42385, Novus Biologicals, USA), mTOR (Catalogue number: RTFI01278, AssayGenie, Ireland), Runx2 (Catalogue number: abx255979, abbexa, UK), OSX (Catalogue number: MBS020109, MyBioSource, USA), OPN (Catalogue number: KA0312, Novus Biologicals, USA), OCN (Catalogue number: NBP2‐68153, Novus Biologicals, USA), LC3 (Catalogue number: RTEB1503, AssayGenie, Ireland), ATG5 (Catalogue number: RTEB1055, AssayGenie, Ireland), BECN1 (Catalogue number: NBP2‐69960, Novus Biologicals, USA), and P62 (Catalogue number: NBP2‐61300, Novus Biologicals, USA).

### 2.2 Cell Isolation and In Vitro Culture

Neonatal rats were used to isolate BMSCs according to an approach previously described by Shim et al. (2016). Isolated cells were then resuspended at a 37°C humidified atmosphere and 5% CO_2_ in DMEM high glucose supplemented with penicillin, FBS, and streptomycin.

### 2.3 Cell Viability Assay

BMSCs were assessed for viability using the MTT (3‐[4,5‐dimethylthiazol‐2‐yl]‐2,5‐diphenyl tetrazolium bromide) method. The protocol involved incubating 1 mL of MTT solution (1 mg/mL) in all plates within a humidified atmosphere containing 5% CO_2_ at 37°C for 5 h. Then, MTT was removed, and each plate received 1 mL of DMSO and was incubated for 1 h. The conversion of the color of the blue formazan dye was finally assessed at a wavelength of 490 nm using a multi‐plate reader.

### 2.4 Knockdown and Overexpression Assays

Specific mimics were purchased from GenePharma (Shanghai, China) to overexpress miR‐125b‐5p. Moreover, Oligoengine software and nucleotide BLAST searches were used to design two SIRT3 siRNA sequences. Transfection was performed when isolated primary osteoblasts were washed with medium and incubated for 6 h. Subsequently, miR‐125b‐5p mimics or control vectors and siRNAs were separately mixed for 5 min with serum‐free medium and incubated for 18 min at 25°C. Finally, the mixture was added to the cells and incubated for 24 h at 37°C.

### 2.5 Experimental Design and In Vivo Study

Twenty‐four female Sprague Dawley rats, 8 weeks old with 220–240 g weights were provided by the Animal Center at the University. Animals were acclimated for 1 week in a controlled laboratory environment (12‐h light/dark cycle, well‐ventilated room, 22°C–25°C temperature, and access to standard food and water *ad libitum*). After acclimatization, rats were randomly allocated to four groups, including CON (controls, received normal saline administration), DEX (injection of intramuscular DEX at a dosage of 1 mg per kilogram daily for 60 consecutive days), LUT50 (received DEX and 50 mg/kg/day luteolin orally), and LUT100 (received DEX and 100 mg/kg/day luteolin orally). Finally, animals were euthanized (by the injection of xylazine 2% and ketamine anesthesia), and the cardiac puncture method was used to collect blood samples, followed by serum separation. In addition, cartilage‐free and bone marrow‐free tibia from each animal were isolated and stored at −80°C for RNA extraction and real‐time polymerase chain reaction (qRT‐PCR) analysis. For histological and stereological analysis, another tibia from each animal was preserved in 10% formaldehyde.

### 2.6 Histological and Stereological Analysis

The samples preserved in 10% formaldehyde were used for histomorphological and stereological analysis of bone tissue. In this regard, the removed tissue was fixed in formalin 10%, processed, and blocked in cylindrical paraffin blocks. Thin serial sections, each measuring 5 μm in thickness, were prepared (10 sections per animal) using a microtome and underwent histological staining with hematoxylin and eosin (H&E). A blinded histological analysis of the samples was conducted by a specialist histopathologist. Moreover, the stereological analyses, including the assessment of bone cell density, the number of bone cells, bone volume, and density of the bone's trabeculae volume, were performed as Vakili et al. (2021) described previously.

### 2.7 Biochemical Analysis

The MyBioSource kits were employed to quantify the concentrations of calcium (Ca), phosphorus (P), and alkaline phosphatase (ALP), which were then processed through the Mindray BS200 autoanalyzer.

### 2.8 RNA Extraction and Real‐Time Quantitative Polymerase Chain Reaction (RT‐qPCR)

EZ RNA reagent was used to extract RNA from the sample as per the manufacturer's instructions. RNA 260/280 and 260/230 ratios were assessed using the Biotek Nanodrop system. The RNA integrity was further assessed through 1.5% agarose gel electrophoresis. Revert Aid First Strand cDNA Synthesis Kit (Fermentase) was used to synthesize complementary DNA (cDNA) using the purified RNA. Moreover, SYBR Green was applied to perform the qRT‐PCR on the Applied Biosystems 7500 System. Specific primer sequences were used to assess the expression levels of *AMPK*, *β‐actin*, *miR‐125b‐5p*, *SIRT3*, *mTOR*, *Runx2*, *OSX*, *OPN*, *OCN*, *LC3*, *ATG5*, *BECN1*, and *p62/SQSTM1* genes. Measured mRNA levels were normalized using *β‐actin* mRNA levels based on the 2^−ΔΔCt^ method.

### 2.9 The Measurement of Proteins Using ELISA

The protein levels of upstream regulators including SIRT3, AMPK, and mTOR were measured by available ELISA kits. Markers related to the osteocytes (Runx2, OSX, OPN, and OCN) and autophagy (LC3, ATG5, BECN1, and p62/SQSTM1) were analyzed using commercial ELISA kits. The levels of the mentioned markers were measured according to the instructions given by the manufacturer. Both cells and tissue homogenates were used for the measurement of the levels of proteins.

### 2.10 Statistical Analysis

The mean ± the standard deviation was used to describe the obtained results. The Kolmogorov–Smirnov test was employed to assess the normality of the data. The Student's *t*‐test and one‐way analysis of variance (ANOVA) were used for conducting the comparisons between groups, followed by a post hoc Tukey test. SPSS software (Windows version 23.0) was used to perform statistical analyses, and graphs were designed by GraphPad Prism 8. A *p*‐value < 0.05 was deemed statistically significant.

## 3 Results

### 3.1 The Effects of DEX and LUT on Cell Viability

**FIGURE 1:**
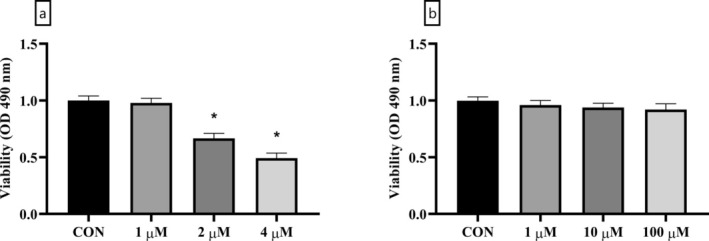
The analysis of BMSC viability by MTT assay. The viability of BMSC after treatment with different concentrations of DEX (a) and LUT (b) is depicted. *: A significant difference with controls (CON). *p*‐value < 0.05 was considered significant.

The MTT method was used in the present study to evaluate the isolated cells' viability after treatment with DEX and LUT at different doses. The findings showed that DEX concentration‐dependently decreased cell viability (Figure 1a). In this regard, a 1 μM concentration of DEX did not induce a significant change in cell viability, although 2 μM and 4 μM doses of DEX significantly decreased the viability of BMSCs (*p*‐value < 0.01). This is even though none of the doses of 1 μM, 10 μM, and 100 μM of LUT had decreased BMSCs viability (as illustrated in Figure 1b, *p*‐value > 0.05). According to the results of the present study as well as previous similar studies (Li et al. 2022, 2020), the doses of 4 μM of DEX and 100 μM of LUT were determined for subsequent in vitro investigations.

### 3.2 LUT Reversed the DEX's Effect on Osteogenesis and Altered the miR‐125b‐5p and SIRT3/AMPK/mTOR Expression

**FIGURE 2:**
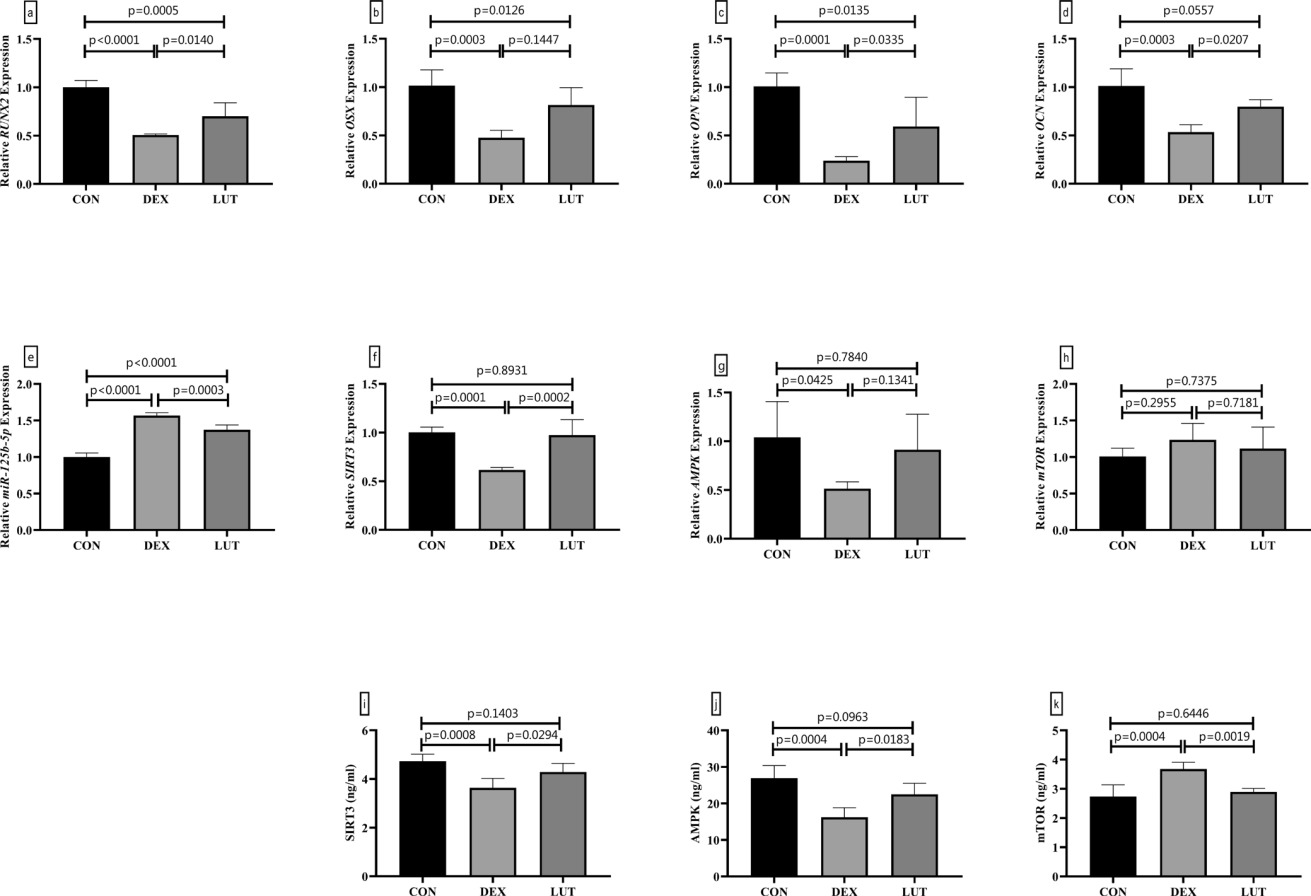
Effects of DEX and LUT on BMSC osteogenesis, miR‐125b‐5p expression, and SIRT3/AMPK/mTOR axis. The administration of LUT restored the levels of RUNX2 (a), OSX (b), OPN (c), and OCN (d). Moreover, altered levels of miR‐125b‐5p expression (e), as well as gene expression and protein levels of SIRT3 (f, i), AMPK (g, j), and mTOR (h, k) were obtained after DEX and LUT treatment. *p*‐value < 0.05 was considered significant.

The RT‐qPCR method was used to investigate the expression of different genes participating in BMSCs differentiation and osteogenesis (Figure 2). The findings showed that the DEX treatment led to a significant reduction in the expression of genes *RUNX2* (49.50%), OSX (53.06%), *OPN* (76.39%), and *OCN* (47.23%) (*p*‐value < 0.001). Additionally, DEX caused a significant elevation in the expression of miR‐125b‐5p (1.57 times) and a significant reduction in the expression of *SIRT3* (38.52%) and *AMPK* (50.58%) genes (*p*‐value < 0.001), while the *mTOR* expression gene did not change after treatment with DEX (*p*‐value = 0.2955). The analysis of SIRT3, AMPK, and mTOR protein levels using the ELISA approach showed that DEX had decreased the levels of SIRT3 and AMPK proteins, while the level of mTOR had significantly increased (*p*‐value < 0.001). According to the injection of LUT, compared to DEX‐treated cells, the expression of *OPN, OSX, RUNX2*, and *OCN* genes was increased, and the expression of miR‐125b‐5p was suppressed (*p*‐value < 0.05). In addition, the injection of LUT revived the expression of SIRT3, AMPK, and mTOR at the gene and protein levels (*p*‐value < 0.05).

### 3.3 LUT Activated Autophagic Flux in DEX‐Treated Cells

**FIGURE 3:**
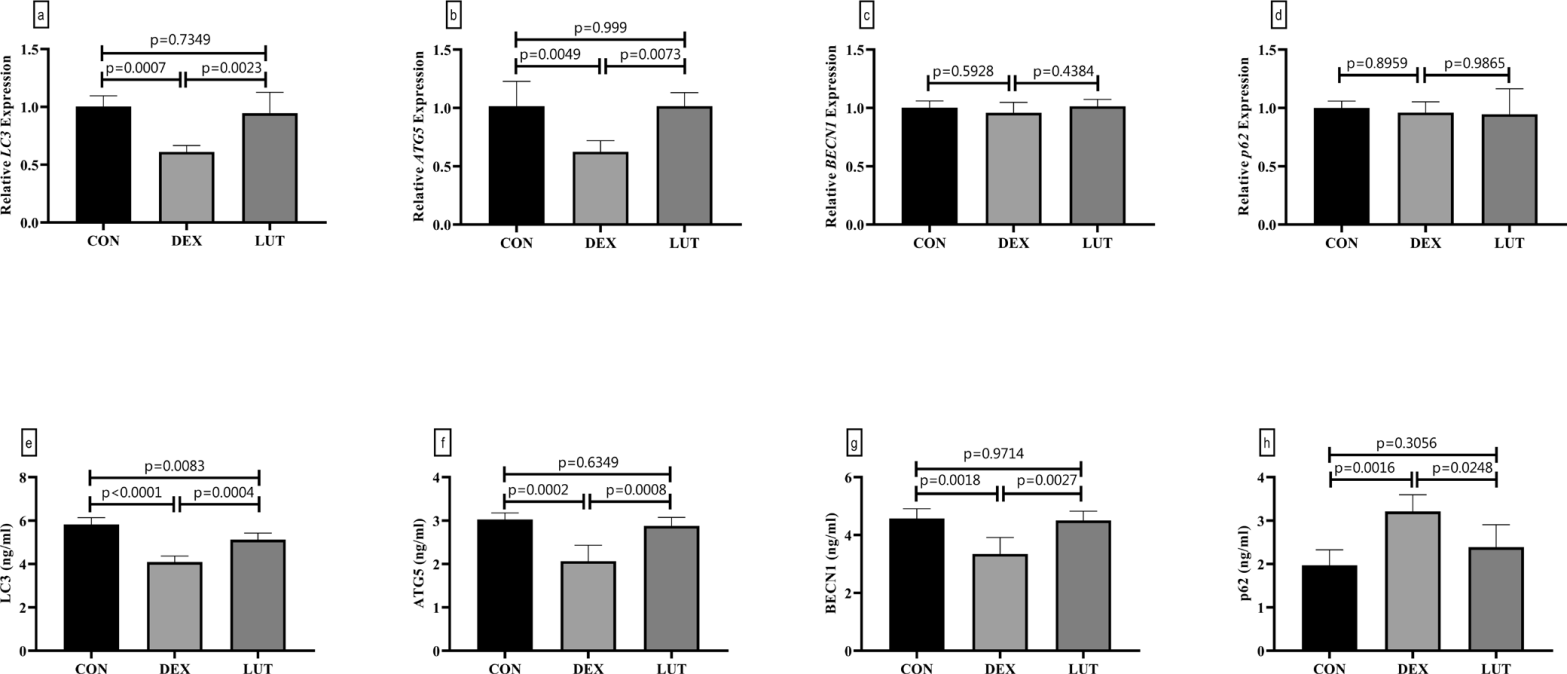
LUT improved autophagic flux in DEX‐treated BMSCs. The gene expression and protein levels of LC3 (a, e), ATG5 (b, f), BECN1 (c, g), and p62 (d, h) were measured using RT‐qPCR and ELISA techniques. *p*‐value < 0.05 was considered significant.

*SIRT3* downstream genes, *AMPK* and *mTOR*, are considered upstream regulators of autophagic flux; therefore, the present study examined major markers of autophagy at the level of gene and protein expression (Figure 3). The findings showed that administration of DEX to BMSCs resulted in a significant decrease in the expression of *LC3* (39.24%, *p*‐value = 0.0007) and *ATG5* (38.58%, *p*‐value = 0.0049) genes, although no change was found in the expression of *BECN1* (*p*‐value = 0.5928) and *p62* (*p*‐value = 0.8959) genes. Moreover, in DEX‐treated BMSCs, a significant reduction in the level of BECN1, ATG5, and LC3 was observed, while the level of p62 protein was upregulated significantly (*p*‐value < 0.001). Importantly, LUT significantly restored the aforementioned changes (*p*‐value < 0.05).

### 3.4 The Effects of LUT Were Reversed by miR‐125b‐5p Upregulation or SIRT3 Downregulation

**FIGURE 4:**
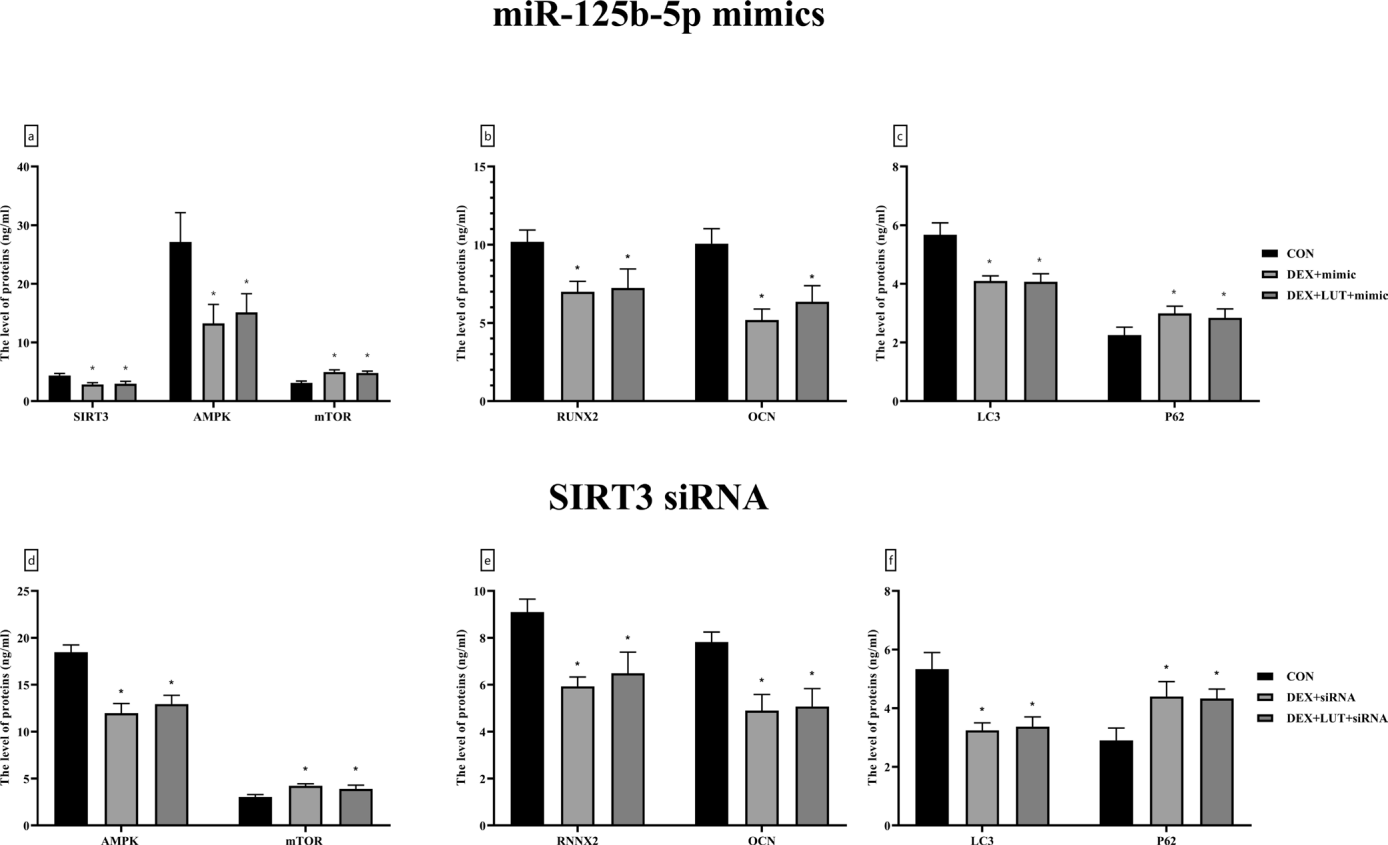
miR‐125b‐5p overexpression and SIRT3 downregulation inhibited therapeutic properties of LUT on DEX‐treated BMSCs. Specific mimics were used to overexpress miR‐125b‐5p and assess the effects of DEX and LUT on SIRT3/AMPK/mTOR axis (a), bone markers (b), and autophagic flux (c) in BMSCs. Moreover, SIRT3 expression was inhibited using specific siRNA, and the levels of AMPK/mTOR (d), bone (e), and autophagy (f) markers were evaluated. *: A significant difference with controls (CON). *p*‐value < 0.05 was considered significant.

Previously, microarrays were considered pivotal regulators of BMSC differentiation and osteogenesis, which play this key role by modulating the expression of downstream pathways (Peng et al. 2016; Zhang et al. 2021). In studies, SIRT3 has been hypothesized to be a regulator of the process of osteogenesis (Huang et al. 2022), whose expression is regulated by miRNAs. Therefore, the present study, in the previous investigations, showed that LUT was able to improve OP caused by DEX treatment by changing the expression levels of SIRT3, AMPK, mTOR, and miR‐125b‐5p and also inducing autophagy. To clarify the effect of LUT through miR‐125b‐5p and downstream regulators, the present study focused on the upregulation of miR‐125b‐5p and the knockdown of SIRT3. The findings revealed that the administration of the miR‐125b‐5p mimic suppressed the ability of LUT to change in the level of regulatory proteins (SIRT3, AMPK, and mTOR, Figure 4a), osteogenesis markers (RUNX2 and OCN, Figure 4b), and autophagic markers (LC3 and P62, Figure 4c) (*p*‐value < 0.01 CON group compared and *p*‐value > 0.05 comparing to DEX + mimic group). Similarly, downregulating SIRT3 expression through specific siRNAs caused LUT's effect on downstream regulators, osteogenesis markers, and the autophagic pathway to be not significantly different from DEX‐treated cells (Figure 4d–f).

### 3.5 LUT Ameliorated the Histomorphology and Bone Biochemistry of DEX‐Treated Animals

**FIGURE 5:**
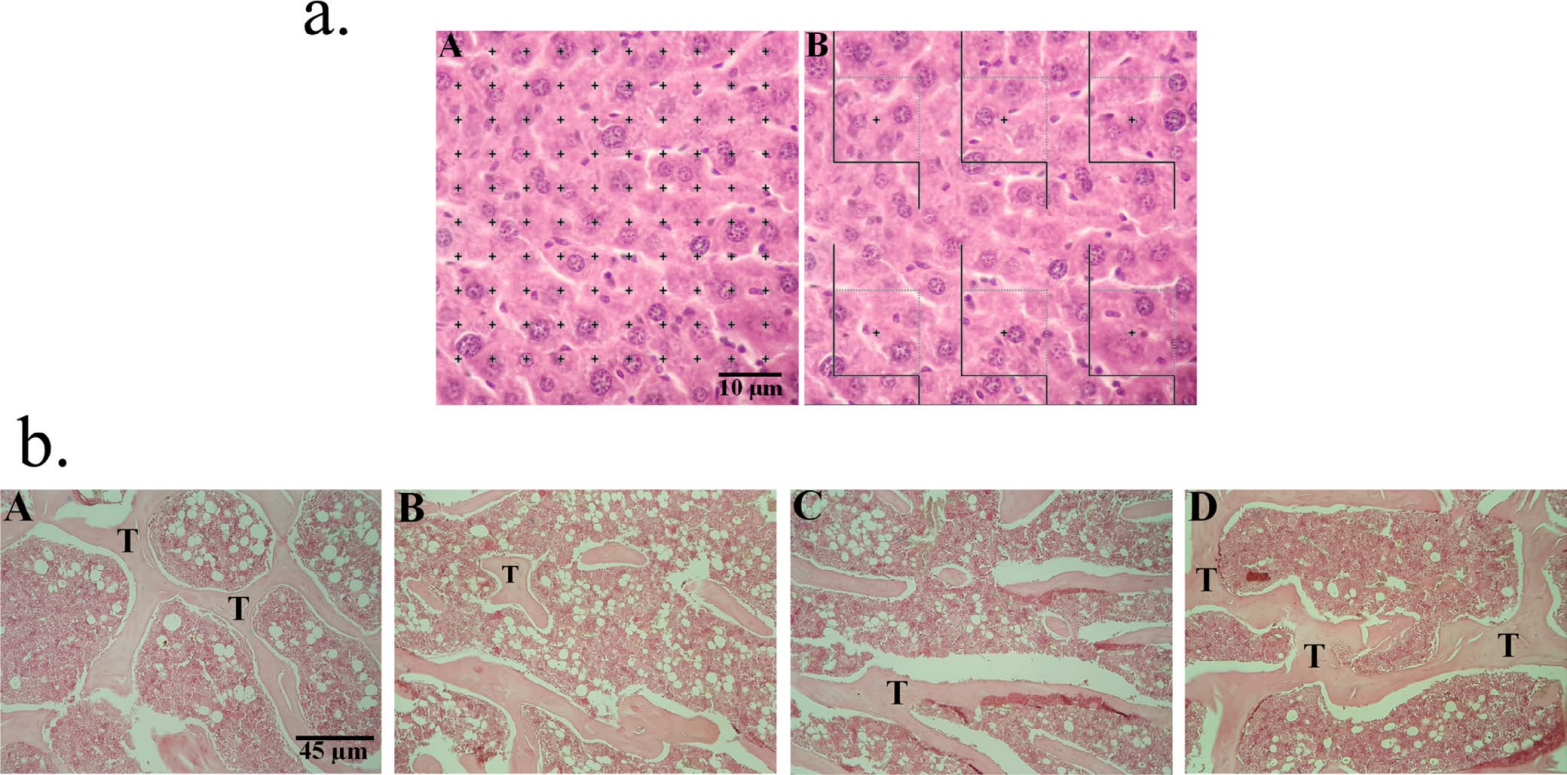
The histological and stereological analysis of bone tissue. (a) an example of the method used for stereological analysis. (b) the histology of bone tissue in CON (A), DEX (B), LUT50 (C), and LUT100 (D) animals. H&E staining was used. Osteoporosis was induced in DEX animals, while LUT at 100 mg/kg/day significantly improved the histoarchitecture of bone tissues.

LUT administration was performed in the rat model of DEX‐induced osteoporosis to analyze the therapeutic effects in the in vivo environment. For this purpose, histological and stereological examinations and biochemical analyses of the isolated bone tissue were performed according to a previously described method (Figure 5a). Histological studies showed that the dose of 100 mg/kg of LUT significantly preserved the normal bone morphology and prevented the manifestations related to OP (Figure 5b). Moreover, the dose of 100 mg/kg of LUT caused a significant increase in the tibia weight (1.44 times), trabecular volume (1.56 times), number of osteocytes (2.30 times), and number of osteoblasts (2.13 times), and also a significant decrease in the number of osteoclasts (63.40%) compared to the DEX group (Table 1). In addition, the dose of 100 mg/kg of LUT significantly increased the level of Ca (1.30 times) and P (1.25 times), while it caused a decrease in the level of ALP (36.50%) compared to the DEX group (*p*‐value < 0.001). Importantly, the animals that were treated with a dose of 100 mg/kg of LUT showed a notable difference with the CON group regarding tibia weight, trabecular volume, number of osteoclasts, and ALP level (*p*‐value < 0.05).

**TABLE 1:** LUT ameliorated bone stereology and biochemistry in animals with DEX‐induced osteoporosis.

Parameters		CON	DEX	LUT50	LUT100
Stereological	Weight of tibia (mg)	393.38 ± 10.18	247.77 ± 5.61*	307.72 ± 4.65**	356.97 ± 4.86**
	Trabecular vol (mm^3^)	146.43 ± 3.83	84.93 ± 4.27*	125.34 ± 4.31**	132.16 ± 2.91**
	No. osteocytes (10^6^)	31.51 ± 2.19	12.61 ± 1.24*	22.43 ± 1.20**	29.03 ± 1.33***
	No. osteoclasts (10^3^)	89.75 ± 5.47	378.36 ± 6.87*	231.46 ± 23.38**	138.49 ± 10.99**
	No. osteoblasts (10^6^)	6.06 ± 0.73	2.65 ± 0.37*	3.90 ± 0.59**	5.65 ± 0.68***
Biochemical	Ca (mg/dL)	8.25 ± 0.52	5.80 ± 0.47*	7.01 ± 0.37**	7.55 ± 0.33***
	P (mg/dL)	7.32 ± 0.65	5.15 ± 0.52*	5.95 ± 0.85**	6.46 ± 0.91***
	ALP (mg/dL)	219.31 ± 10.32	426.54 ± 3.62*	396.83 ± 4.08**	270.85 ± 32.15**

* Significant difference with CON.

** Significant difference with CON and DEX.

*** Significant difference with DEX.

### 3.6 LUT Altered the miR‐125b‐5p Expression and Downstream Regulators

**FIGURE 6:**
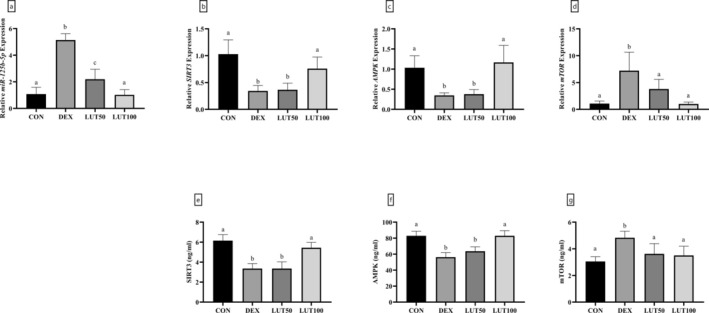
The expression of miR‐125b‐5p and SIRT3/AMPK/mTOR axis in animals with OP. DEX‐treated animals revealed overexpressed levels of miR‐125b‐5p (a). The gene expression and protein levels of SIRT3 (b, e) and AMPK (c, f) significantly reduced in the DEX group, while mTOR (d, g) was remarkably upregulated. LUT at a dose of 100 mg/kg/day ameliorated DEX‐induced alterations in miR‐125b‐5p expression and SIRT3/AMPK/mTOR axis. Similar lowercase letters on bars represent no significant difference between groups. *p*‐value < 0.05 was considered significant.

Gene expression analysis using the RT‐qPCR method showed that the administration of DEX in rats was accompanied by a significant increase of 4.75 times in the expression of miR‐125b‐5p compared to the controls (Figure 6, *p*‐value < 0.001), which was followed by a significant decrease in the expression of genes encoding *SIRT3* (66.61%) and *AMPK* (66.28%). On the contrary, the expression of the gene encoding *mTOR* had a significant increase of 6.69 times when the DEX group was compared with the controls (*p*‐value < 0.001). Moreover, the examination of the levels of SIRT3, AMPK, and mTOR proteins in the DEX and control groups showed a significant change (respectively, a 45.43% and 32.15% decrease and a 1.58‐fold increase in the levels of SIRT3, AMPK, and mTOR, *p*‐value < 0.01). Importantly, the injection of a dose of 100 mg per kg of LUT caused the effects of DEX on the expression of miR‐125b‐5p and also gene and protein expression levels of SIRT3, AMPK, and mTOR to be notably restored (*p*‐value < 0.05).

### 3.7 LUT Improved Osteogenesis in Rats With Osteoporosis

**FIGURE 7:**
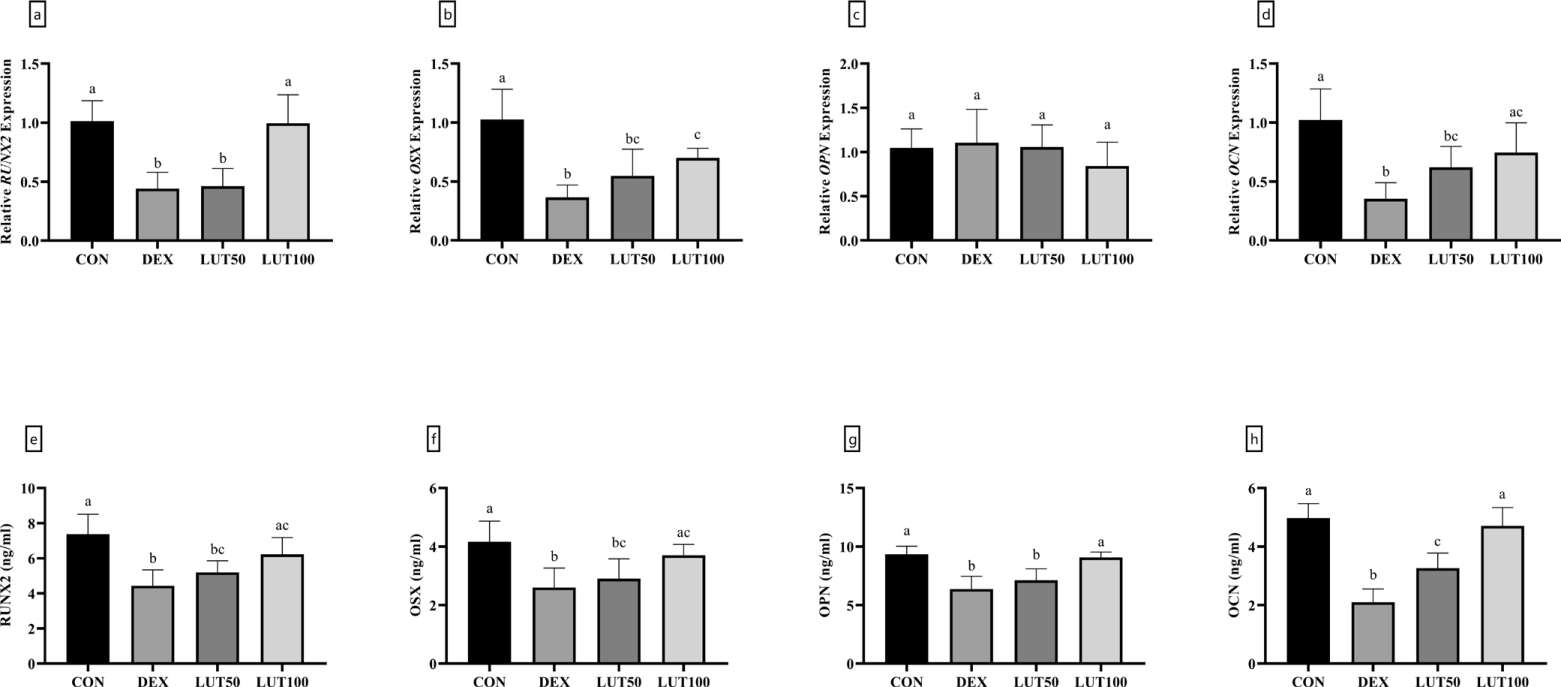
Effects of DEX and LUT on osteogenesis markers. The administration of LUT restored the gene expression and protein levels of RUNX2 (a, e), OSX (b, f), OPN (c, g), and OCN (d, h). Similar lowercase letters on bars represent no significant difference between groups. *p*‐value < 0.05 was considered significant.

Analysis of osteogenesis was performed by molecular examination of related factors utilizing ELISA and RT‐qPCR techniques. Comparing the OP model caused by DEX with the controls, it was determined that the gene expression and protein levels of RUNX2 (56.40% and 39.92% decrease in the gene and protein expression levels, respectively), OSX (64.46% and 37.62% reduction in the gene and protein expression levels, respectively), and OCN (65.36% and 57.85% decrease in the gene and protein expression levels, respectively) were significantly decreased (*p*‐value < 0.0001, Figure 7). Meanwhile, administering a dose of 100 mg/kg of LUT caused the gene expression and level of RUNX2, OSX, and OCN proteins to increase significantly compared to the DEX group (*p*‐value < 0.001). Interestingly, the *OPN* gene expression showed no statistically significant variation between the examined groups (*p*‐value = 0.328); however, a notable reduction in the protein levels of OPN was found in the DEX group compared to the controls (6.37 ng/mL in DEX animals vs. 9.35 ng/mL in CON group, *p*‐value < 0.001). Nevertheless, the injection of a dosage of 100 mg/kg of LUT was able to significantly elevate the level of OPN protein compared to the DEX group (*p*‐value < 0.01).

### 3.8 Autophagy Was Activated by LUT Treatment in Osteoporosis Animals

**FIGURE 8:**
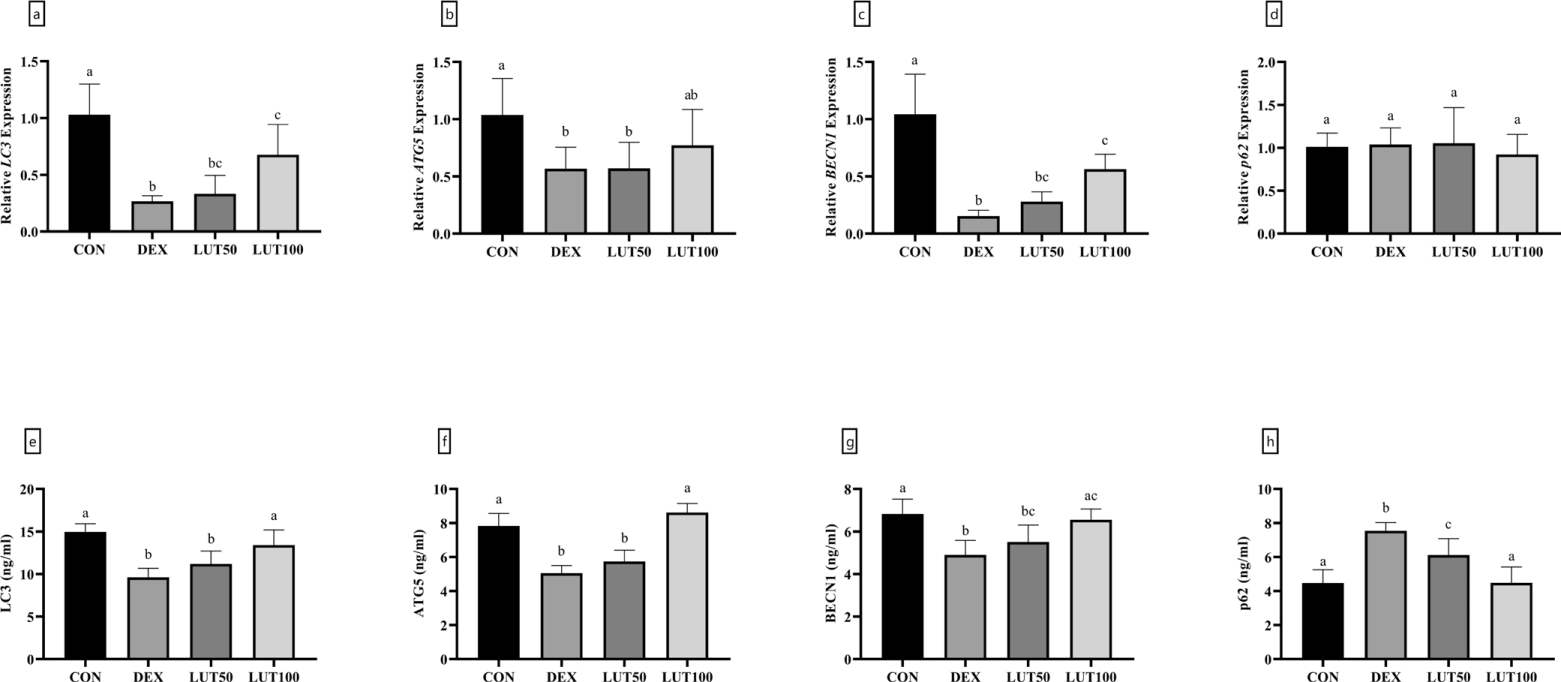
LUT enhanced autophagic flux in DEX‐treated animals. The gene expression and protein levels of LC3 (a, e), ATG5 (b, f), BECN1 (c, g), and p62 (d, h) were analyzed. Similar lowercase letters on bars represent no significant difference between groups. *p*‐value < 0.05 was considered significant.

The analysis of autophagy‐related markers revealed that in the DEX group, compared to the controls, the levels of LC3, ATG5, and BECN1 decreased significantly in terms of gene expression (74.11%, 45.35%, and 85.29% decrease, respectively) and protein (35.76%, 35.51% and 28.20% decrease, respectively) levels (Figure 8). This is even though the administration of 100 mg/kg of LUT had significantly restored the levels of LC3, ATG5, and BECN1 markers in terms of gene and protein expression in comparison with the DEX group (*p*‐value < 0.01). Interestingly, the gene expression of *p62* did not reveal any notable difference between the studied groups (*p*‐value = 0.8363). Although there was a significant elevation in the DEX (1.69 times) and LUT50 (1.37 times) groups compared to controls (*p*‐value < 0.001), a significant decrease in the LUT100 group compared to the DEX group in terms of p62 protein levels was observed (*p*‐value < 0.001).

## 4 Discussion

As one of the major health issues globally, OP affects thousands of millions and is the most prevalent bone‐related metabolic disorder (Song et al. 2022); however, the underlying mechanism of OP progression and a reliable therapeutic strategy are not elucidated. Recently, miR‐125b‐5p and the SIRT3/AMPK/mTOR axis are recognized as molecular regulators involved in OP occurrence, although the interaction between them has not been investigated. Moreover, LUT is recently described as a flavonoid with possible anti‐osteoporosis properties. The current investigation constructed in vitro and in vivo models of DEX‐induced OP and studied the function of miR‐125b‐5p and the SIRT3/AMPK/mTOR axis in OP occurrence, the therapeutic potential of LUT, and the ability of LUT to modulate miR‐125b‐5p expression, as well as the regulation of the SIRT3/AMPK/mTOR axis and downstream autophagic flux.

The obtained data revealed that the administration of DEX in BMSCs was associated with a significant reduction in the expression markers of osteogenesis and mineralization, including OPN, OSX, RUNX2, and OCN. It has previously been shown that RUNX2 and its downstream transcription factor, OSX (Zhang 2010), have potentially contributed to the differentiation of osteoblasts and bone formation; hence, the absence of these markers led to impaired osteoblastic differentiation and lack of bone mineralization in animal models. Interestingly, the deficiency of RUNX2 and OSX causes the shift of BMSCs, which are considered the origin of both osteocytes and adipocytes, from osteogenesis toward adipogenesis (Ghali et al. 2015; Pierce et al. 2019). Similarly, it has been previously demonstrated that the administration of DEX causes the suppression of osteogenesis and mineralization through the reduction in OPN and OCN levels (He et al. 2024; Jiang et al. 2021). Further in vivo studies by the present investigation showed that RUNX2, OSX, OPN, and OCN gene expression and protein levels were significantly reduced in rats treated with DEX compared to controls, which was accompanied by damage to bone histology. In fact, the results of the current study revealed that the number of osteoblasts and osteocytes had declined remarkably in DEX‐treated animals, whereas a notable increase in the number of osteoclasts was achieved. As the cells lying within the bone, osteocytes are recognized as the main mechanoreceptors (Yang et al. 2023), while osteoblasts originate from osteoprogenitor cells and are responsible for the synthesis of the components that constitute the extracellular matrix of bone and promote mineralization of the organic matrix (Šromová et al. 2023). It is widely suggested that the reduction in osteocytes and osteoblasts count reduction along with the elevation in the number of osteoclasts—cells that initiate bone remodeling or bone loss—are the main histological manifestations of OP (Gossiel et al. 2016; Liu et al. 2017; Wang et al. 2023). Interestingly, current both in vitro and in vivo investigations revealed that the administration of LUT remarkably restored the levels of bone molecular markers altered by DEX treatment. Indeed, a variety of studies have suggested that phytochemicals could prevent the occurrence and progression of OP through different mechanisms, such as mimicking estradiol properties (Shah et al. 2022), altering signaling pathways regulating osteogenesis (Sharma et al. 2023), suppressing regulated cell death programs such as apoptosis (Saleh et al. 2024), and preventing inflammation and oxidative stress (Li et al. 2023). Recent findings from network pharmacology studies, as well as cell and animal investigations, have shown that LUT could promote BMSCS osteogenic differentiation by modulating the activity of the Pi3K‐AKT signaling pathway; hence, preventing ovariectomy‐induced OP (Chai et al. 2024; Liang et al. 2022).

Regulation of BMSC differentiation toward osteogenesis requires complicated signaling processes controlled by upstream effectors including non‐coding RNAs. In fact, several studies have shown that miRNAs (e.g., miR‐26b, miR‐224 and miR125b) regulate the process of osteogenesis through the regulation of downstream axes such as SIRT3/AMPK/mTOR and Pi3K/AKT (Chen and Dai 2023; Zhao et al. 2021). In this regard, it has been determined that miR‐125 promotes OP by regulating downstream modulators such as the TRAF6 and BMPR1 pathways (Ogasawara et al. 2024; Wang et al. 2021, 2017). Moreover, as a member of mitochondrial sirtuins, SIRT3 plays a pivotal role in cell homeostasis and is involved in a variety of aging‐related diseases (Li and Cai 2023). It is demonstrated that the senescence of BMSCs could lead to osteogenic damage and OP, hence SIRT3 is assumed to be involved in the OP occurrence (Guo et al. 2021). Accordingly, the regulation of SIRT3 expression through miRNAs and altered downstream AMPK/mTOR signaling has previously been suggested as contributors to OP (Chen and Dai 2023). The present study showed that in both experimental settings including in vitro and in vivo models, induction of OP is accompanied by an increase in the miR‐125b‐5p expression, which is related to the downregulation of SIRT3 and AMPK and the overexpression of mTOR. Mechanistically, the application of miR‐125b‐5p mimics and SIRT3 siRNA showed that overexpression of the former or suppression of the latter has the same consequences on the markers expression related to bone. In addition, the restorative properties of LUT on DEX‐induced changes in miR‐125b‐5p expression and the SIRT3/AMPK/mTOR axis were inhibited after miR‐125b‐5p overexpression or SIRT3 inhibition. Therefore, one can conclude that miR‐125b‐5p participates in the induction of OP through the SIRT3/AMPK/mTOR axis, while the administration of LUT inhibits the expression of miR‐125 and consequently changes in the downstream SIRT3/AMPK/mTOR axis.

A plethora of evidence has suggested mTOR as a negative regulator of autophagy (Samare‐Najaf, Neisy, et al. 2023; Samare‐Najaf, Samareh, et al. 2023; Kim et al. 2011). Autophagy is a catabolic pathway that provides materials and energy by breaking down redundant intracellular macromolecules and thus plays a role in cell survival. However, under certain conditions, it can appear as a mechanism of programmed cell death (Samare‐Najaf, Samareh, et al. 2023). Previous studies have extensively investigated the role of autophagy in OP progression (Yang et al. 2019; Zheng et al. 2020). The present study revealed that the induction of OP, both in vitro and in vivo, was accompanied by overexpression of mTOR, which in turn was inhibited upon the administration of LUT. Therefore, this investigation aimed to evaluate the level of factors related to autophagy in BMSCs and bone tissue isolated from the animals with OP. The results showed that the administration of DEX was accompanied by the downregulation of LC3, ATG5, and BECN1, while p62 was overexpressed. Considering the role of these proteins in promoting the autophagic process (Samare‐Najaf, Neisy, et al. 2023), it can be concluded that autophagy was suppressed in OP. Indeed, autophagic flux is pivotal to the survival, differentiation, and functioning of bone cells (Yin et al. 2019). Autophagy dysregulation consequently disrupts bone formation/bone resorption balance and mediates the onset and progression of OP (Zhang, Qi, et al. 2020). Interestingly, the ameliorating effects of LUT on OP can also be attributed to the induction of autophagy in bone cells, as different phytochemicals have been suggested as modulators of the autophagic pathway and as a therapeutic strategy for OP (Vakili et al. 2021; Yang et al. 2019). Moreover, it has been shown that autophagy and stress interact; hence, the modulation of the autophagic pathway by phytochemicals may be attributed to their anti‐stress properties (Al Azzani et al. 2024; Pinelli et al. 2023).

The current investigation has established the effectiveness of LUT in both cellular and animal models of OP, elucidating potential mechanisms through methodologies such as histo‐stereological analysis, RT‐qPCR, spectrophotometry, and ELIS. However, additional research is essential to further elucidate these aims. In particular, more comprehensive in vitro and in vivo studies employing techniques such as Western blotting, electron microscopy, flow cytometry, immunohistochemistry, etc. are required to explore the role of the proposed pathway in the OP progression. Furthermore, the validation of LUT's efficacy cannot be achieved solely through animal studies; thus, clinical trials are strongly recommended for future exploration. Based on the findings of this study, should subsequent research yield consistent results, LUT may represent the potential for a safe and effective therapeutic strategy for the treatment of OP.

## 5 Conclusion

The present findings revealed that OP is associated with the miR‐125b‐5p overexpression and alteration in the SIRT3/AMPK/mTOR axis, which is followed by the suppression of autophagy in BMSCs and bone tissue. Meanwhile, the administration of luteolin was able to improve the bone tissue of animals treated with DEX according to histological, biochemical, and molecular analyses. The change in the expression of miR‐125b‐5p and the SIRT3/AMPK/mTOR axis, which was accompanied by the induction of autophagy, could be considered the underlying mechanism of the therapeutic properties of luteolin in confronting OP. Nonetheless, the current findings cannot be assumed to be evidence for the application of luteolin as a therapeutic strategy in patients with OP, since further studies, especially clinical trials, are necessary.

## Author Contributions

**Liang Tang:** conceptualization (equal), formal analysis (equal), methodology (equal), writing – original draft (equal), writing – review and editing (equal). **Xinyu Fan:** formal analysis (equal), methodology (equal), writing – review and editing (equal). **Yongqing Xu:** formal analysis (equal), methodology (equal), writing – original draft (equal). **Yeming Zhang:** formal analysis (equal), methodology (equal), writing – review and editing (equal). Gang Li: conceptualization (equal), writing – review and editing (equal), supervision.

## Ethics Statement

The experimental procedures and animal care and handling were approved by Local Animal Care Ethics. Moreover, all efforts were made to minimize the animals' suffering based on the ARRIVE guidelines.

## Consent

The authors have nothing to report.

## Conflicts of Interest

The authors declare no conflicts of interest.

## Supporting information


Figure S1

## Data Availability

The datasets used and/or analyzed during the current study are available from the corresponding author upon reasonable request.
