# Supplementary material for: Luteolin Inhibits Dexamethasone‐Induced Osteoporosis by Autophagy Activation Through miR‐125b‐5p/SIRT3/AMPK/mTOR Axis, an In Vitro and In Vivo Study
Source: Food Sci Nutr. 2025 Mar 17;13(3):e70071. doi: 10.1002/fsn3.70071 (PMC11913733; doi:10.1002/fsn3.70071)
Supplement: Supplementary file 1 — Figure S1 [file FSN3-13-e70071-s001.docx]

**Luteolin inhibits dexamethasone-induced osteoporosis by autophagy activation through miR-125b-5p/SIRT3/AMPK/mTOR axis, an in vitro and in vivo study**

**Liang Tang, Xinyu Fan, Yongqing Xu, Yeming Zhang, Gang Li***

The current findings revealed that Luteolin (LUT) could promote osteogenesis and improve osteoporosis (OP) via autophagy activation through miR-125b-5p/SIRT3/AMPK/mTOR pathway.

**
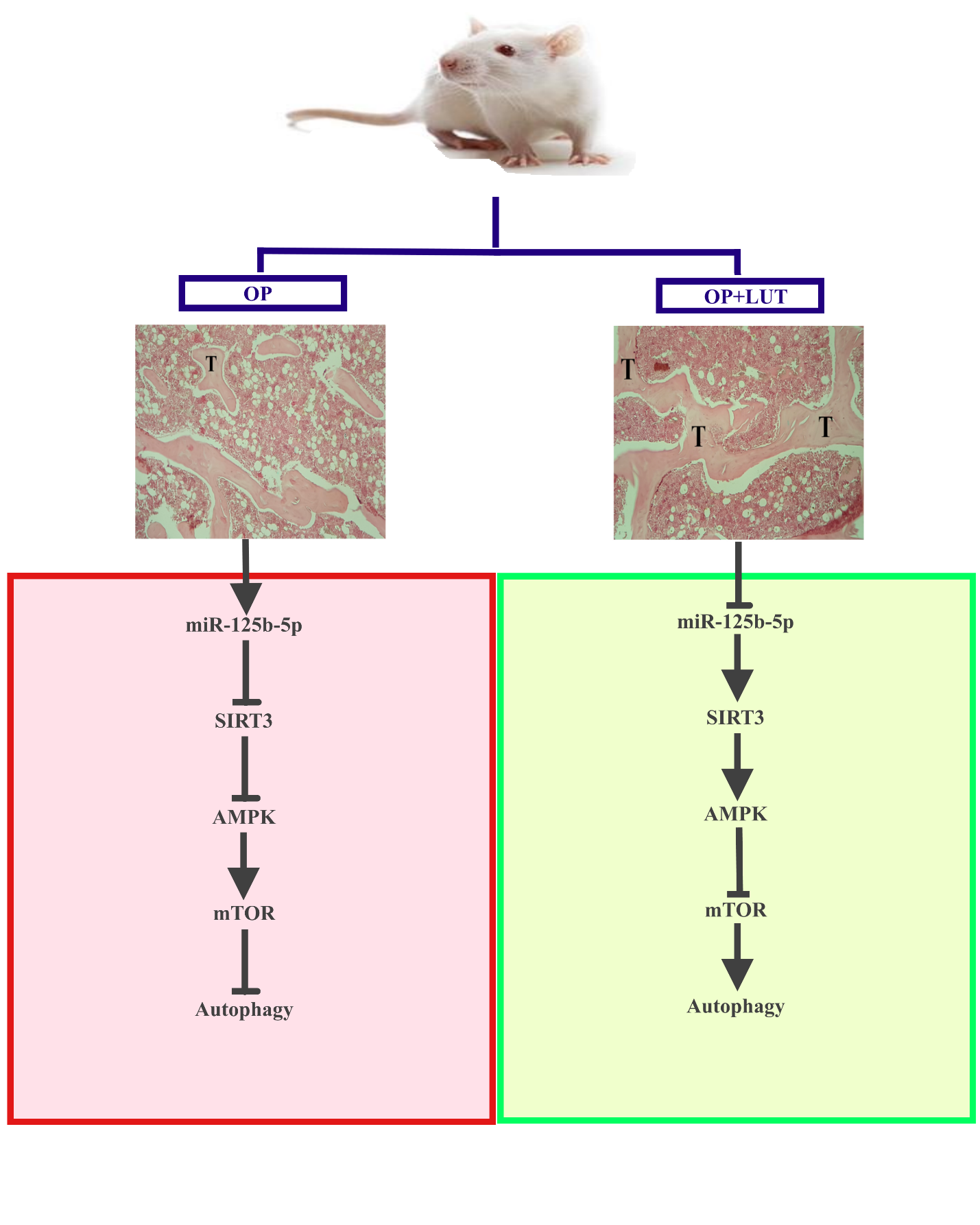
**
